# CT image quality over time: comparison of image quality for six different CT scanners over a six‐year period

**DOI:** 10.1120/jacmp.v16i2.4972

**Published:** 2015-03-08

**Authors:** Ana Maria A. Roa, Hilde K. Andersen, Anne Catrine T. Martinsen

**Affiliations:** ^1^ The Intervention Centre, Oslo University Hospital Oslo; ^2^ The Department of Physics University in Oslo Oslo Norway

**Keywords:** CT, Catphan, image quality, QA

## Abstract

UNSCEAR concluded that increased use of CT scanning caused dramatic changes in population dose. Therefore, international radiation protection authorities demand: 1) periodical quality assurance tests with respect to image quality and radiation dose, and 2) optimization of all examination protocols with respect to image quality and radiation dose. This study aimed to evaluate and analyze multiple image quality parameters and variability measured throughout time for six different CT scanners from four different vendors, in order to evaluate the current methodology for QA controls of CT systems. The results from this study indicate that there is minor drifting in the image noise and uniformity and in the spatial resolution over time for CT scanners, independent of vendors. The HU for different object densities vary between different CT scanner models from different vendors, and over time for one specific CT scanner. Future tests of interphantom and intraphantom variations, along with inclusion of more CT scanners, are necessary to establish robust baselines and recommendations of methodology for QA controls of CT systems, independent of model and vendor.

PACS number: 07, 87

## I. INTRODUCTION

Great developments in CT technology, such as the introduction of spiral scanning, multirow detector systems, exceptionally short scanning times, together with the introduction of new techniques such as organ perfusion, three‐dimensional angiography, and virtual colonography, have made CT an increasingly popular diagnostic tool.^1^, ^2^, ^3^ Recent data from the Organization for Economical Co‐operation and Development^(4)^ and the IMV Medical Information Division^(5)^ show that 13.4 million more CT examinations were performed in 2011 compared to 2009 in the United States. In Norway, the number of CT examinations performed has increased from 6.5% of the total radiological examinations in 1993 to 20% in 2012.^(6)^ CT scans result in higher radiation dose to patients, in comparison to other radiological examinations. With the increased utilization of CT as a diagnostic tool, the attention concerning radiation exposure has also been intensified. The Norwegian Radiation Protection Authority (NRPA) encourages radiology departments to focus on optimization and justification of medical examinations involving ionizing radiation.^(6)^ Moreover, the NRPA demands this optimization of both radiation dose and image quality, by aiming for a balance between the benefits and risks of the examination in accordance to the ALARA (As Low As Reasonably Achievable) principle^1^, ^6^ in order to reduce stochastic effects of radiation, such as cancer induction. Yet, an intrinsic problem of reducing radiation dose in CT examinations is magnification of noise and thereby loss of signal. However, modern CT technology includes advanced techniques for image reconstruction and dose reduction. During the last 30 years, manufacturers have developed new reconstruction techniques and several postprocessing tools to improve image quality. In spite of still being power‐ and potentially time‐consuming, iterative reconstruction methods appear to improve image quality and thereby give potential for dose reduction.^1^, ^7^, ^8^, ^9^ The image quality and radiation dose is affected by the detector system, output from the X‐ray tube, and the image reconstruction techniques, among other factors. Technical quality assurance tests for computed tomography are necessary to ensure that the system is fulfilling its technical specifications. International radiation protection authorities recommend and also demand that quality assurance tests of radiological equipment be performed periodically. Further, which testing methods and parameters to be tested for different types of equipment are suggested in recommendations.^10^, ^11^, ^12^, ^13^, ^14^, ^15^, ^16^, ^17^, ^18^, ^19^, ^20^, ^21^, ^22^, ^23^ The aim of annual quality assurance test is to ensure that the quality of the production of medical images provide the right information.^(24)^ In 2012, the Nordic Association for Clinical Physics (NACP) proposed a protocol for quality assurance (QA) of diagnostic computed tomography scanners,^(25)^ in which remedial and suspension tolerance levels for QA tests were established in accordance to international guidelines and vendor specifications as an effort to standardize these limits in the Nordic countries. However, standardization of QA protocols for computer tomography is a highly challenging task because of the different parameter settings varying from CT scanner model to model and also between vendors. Moreover, the international guidelines become rapidly outdated due to the fast development and incorporation of new scanner models with different parameter settings. Therefore, it is necessary to optimize both tolerance levels and test frequency for each of the tests in a QA program by analyzing the results from acceptance and constancy tests throughout lifetime of different CT systems.

The aim of this study was to evaluate and analyze various image quality parameters and variability measured throughout time for six different CT scanners from four different vendors, to evaluate the current methodology for QA controls of CT systems.

## II. MATERIALS AND METHODS

The tests performed in this study are based on recommendations from The International Electrotechnical Commission (IEC), Institute of Physics and Engineering in Medicine (IPEM,) and The American Association of Physicists in Medicine (AAPM).

### A. The Catphan phantom

Catphan 500/600 is especially designed for evaluation of various image quality parameters in CT, and is used worldwide for image quality assurance testing.^25^, ^26^, ^27^ In this study, four different Catphan phantoms (The Phantom Laboratory, Salem, NY) were used for all image quality tests (one Catphan 500, one Catphan 504 and two Catphan 600s). The Catphan phantom is a cylindrical phantom of around 20 cm of length (depending on model) and a diameter of 20 cm, consisting of different test modules. The image quality tests evaluated in this study were slice thickness (module CTP401 or CTP 404); spatial resolution (CTP528 module); uniformity and noise (module CTP486); and linearity and CT number (module CTP401 or CTP 404). Each test and the corresponding Catphan module used in this study are summarized in Table 1. The three Catphan models utilized in the measurements are quite similar; however, they vary in the module for testing the CT number of different materials by the number of sensitometric inserts of different materials, which is four in Catphan 500 (air, teflon, acrylic, and LDPE) and six in both Catphan 504 and 600 (same materials as in Catphan 500, plus Delrin and PMP). Another difference between the models is the absence of a point source used for automatical measurement of the spatial resolution in Catphan 504. The CT scanners used in this study were placed in different radiology departments, owning their own Catphan phantom, so the phantoms normally used in each department were used in this study.

**Table 1:** Image quality parameters tested including the corresponding test modules of the Catphan phantom

*Parameter*	*Catphan Module*
Slice thickness	CTP401^a^/ CTP404^b^
Spatial resolution	CTP528
Uniformity and noise	CTP486
Linearity and CT number of different materials	CTP401^a^/ CTP404^b^

^a^ In Catphan 500.

^b^ In Catphan 504 and 600.

### B. CT scanners

The results from acceptance tests and annual quality assurance tests for six different CT scanners of different models and from four different vendors, as presented in Table 2, were utilized in this study. The results from these quality assurance tests have been gathered in a database from which the information on image quality used in this study has been collected. All CT scanners included in the study were placed in the same hospital, but in different radiology departments. The hospital has service agreements for all of them, and all units were serviced regularly. As far as possible, all tests presented in this study were performed after the regular preventative maintenance. The workload on each scanner was in the same order of magnitude, except for the Philips Brilliance 64 that had heavier workload compared to the others. All the scanners in the study were used for general CT diagnostics in addition to cardiac and neuroimaging.

**Table 2:** CT scanners used in this study

*Manufacturer*	*Model*	*Slices*	*Abbreviation*
GE Medical Systems, Milwaukee WI, US	Lightspeed 16	16	GE L16
GE Medical Systems, Milwaukee WI, US	Lightspeed VCT	64	GE VCT
Toshiba Medical Systems, Tokyo, Japan	Aquilon One	64	T Aq 1
Philips Medical Systems, Best, The Netherlands	Brilliance 16	16	PB 16
Philips Medical Systems, Best, The Netherlands	Brilliance 64	64	PB 64
Siemens Medical Solutions, Erlangen, Germany	Somaton Sensation	64	SSS 64

### C. Image acquisition protocols

The CT image acquisition protocols for the quality assurance tests used in our hospital were revised and standardized for all scanners in 2010. Therefore, data from quality assurance tests performed before this standardization were excluded, with exception of those tests where the protocols utilized were identical to the standardized ones. The test frequency varied between different units before the test revision and test standardization due to different practice in different departments. All CT scanners were tested at least once a year.

In Table 3, all scan parameter settings used in the study are listed. SFOVs used are head and body, since these FOVs are available for all CT scanners. For some CT scanners it is possible to adjust the SFOV, while others have virtual SFOV, meaning that the SFOV is 500 mm independent of planned SFOV. DFOV was chosen to fit the phantom size for all scanners. For helical scans body SFOV and pitch near 1 was used.

**Table 3:** Acquisition parameters used for measurements of uniformity and image noise

*Technique*	*Axial and Helical*
kVp	120
mAs	250
Rotation time	1 s
Reconstruction kernel	Abdominal^a^
SFOV	Head and Body/Large Body (axial scans)
	Body/Large Body (helical scans)
DFOV	210 mm
Matrix	512×x512
Pitch (helical scans)	As near 1 as possible

^a^ Corresponding filters were Standard for both GE VCT and GE Lightspeed 16, FC13 for Toshiba Aquilon One, B31s, H31s and B30f for the Body, Head, and Helical uptakes in Siemens Somaton Sensation, respectively, and B for both Philips Brilliance 16 and 64.

#### C.1 Uniformity and noise

The module CTP486 was used for measurement of uniformity and image noise, in accordance to the protocol described in Table 3. Due to variability in the parameter settings of different CT scanners, it was necessary to define the slice thickness for each of these scanners, as shown in Table 4. In order to measure uniformity, five regions of interests (ROIs) of $100\;{\text{mm}}^{2}$ were placed in the image, one in the center and four in the periphery, at 0°, 90°, 180°, and 270°, clockwise. The uniformity value was defined as

(1) $$\Delta \text{HU}={\text{HU}}_{\text{max}}={\text{HU}}_{\text{min}}$$

where ${\text{HU}}_{\text{max}}$ is the mean HU value in the ROI with the highest average value, and ${\text{HU}}_{\text{max}}$ is the mean HU value in the ROI with the lowest average value of the five ROIs. The advised limit for HU in water is ΔHU≤4.^(10)^ Measured deviations in image noise compared to established baselines from acceptance test should be within 10%.^(14)^ The deviations in image noise between the image slices within the same total collimation should be less than 10% from the mean of all image slices.^(14)^

Image noise was quantified by measuring the standard deviation (SD) in HU in a circular ROI that covers 40% of the diameter of the homogeneous module of a Catphan phantom in all the slices in this module,^(10)^ with the exception of the Toshiba Aquilion ONE scanner, where noise was measured in the same manner but in the water phantom supplied by the manufacturer, to cover the full detector collimation.

The analysis of the image noise was performed in two ways: 1) the average noise (SD) for each scanner was presented as an absolute value in HU and shown as a function of time, and 2) the noise values were normalized with respect to the slice thickness and radiation dose in order to compensate for the differences in available slice widths and CTDI for each scanner, and the results were time‐averaged to give a better overview of how this parameter varies for each scanner. The normalized noise SDN was calculated as

(2) $$SD_{N}=SD_{A}\sqrt{\frac{1}{CTDI_{ST}}\frac{ST}{ST_{N}}}$$

where ${\text{SD}}_{A}$ is the measured absolute noise, ${\text{CTDI}}_{\text{ST}}$ is the CT dose index for the slice thickness ST used in the noise measurements, and ${\text{ST}}_{N}$ is the slice thickness to which the noise is to be normalized, in this case 1.5 millimeters.

**Table 4:** Reconstructed slice thickness for the measurement of uniformity and noise

*Technique*	*Axial Body*	*Axial Head*	*Helical*
*CT Scanner*	*Slice thickness (mm)*		
GE VCT	2.5	2.5	5.0
GE Lightspeed 16	1.25 1	1.25	5.0
Toshiba Aquilon ONE	2.0	2.0	3.0
Philips Brilliance 16	1.5 1	1.5	5.0
Siemens Somaton Sensation 64	2.4	2.4	5.0
Philips Brilliance 64	0.625	0.625	5.0

#### C.2 Linearity and CT number in different materials

Linearity and CT numbers were measured in the CT images acquired from module CTP401 or CTP404 of the Catphan phantoms 500, 504, or 600, using the scan protocol described in Table 5. The module CTP401 of Catphan 500 contains sensitometric targets made of teflon, acrylic, low‐density polyethylene (LDPE), and air, whereas Catphan 504 and 600 consists of all the previous materials, in addition to delrin and polymethylpentene (PMP).

The CT number in each sensitometric insert was measured by calculating the average HU in a circular ROI with a diameter of 5 mm, drawn on each insert in the CT image. There are differences in beam energy between different CT scanners. Still, the linearity was calculated by finding the Pearson correlation between the measured CT number value and the linear attenuation coefficients in each of the materials at energy of 60 keV since this value is assumed to correspond to the average energy of the X‐ray spectrum after filtration when the input energy is 120 kVp.

**Table 5:** The acquisition parameters used for measurement of linearity and CT numbers

*Technique*	*Axial and Helical*
kVp	120
mAs	250
Rotation time	1 s
Reconstruction kernel	Abdominal^a^
Detector collimation	Max total collimation
SFOV	Body/Large body/Head
DFOV	210 mm
Matrix	512×512
Reconstructed slice thickness^b^ (mm)	2.5−3.0 and 5

^a^ Corresponding filters were Standard for both GE VCT and GE Lightspeed 16, FC13 for Toshiba Aquilon One, B31s, H31s and B30f for the Body, Head, and Helical scans in Siemens Somaton Sensation, respectively; and B for both Philips Brilliance 16 and 64.

^b^ The reconstructed slices for this test in Toshiba Aquilon One had a nominal thickness of 4.0 mm for the axial scans and 3.0 mm for the helical scans.

#### C.3 Spatial resolution

For this test, images were taken in the module CTP528 of a Catphan 500 or 600 phantom, where a tungsten carbide bead with a diameter of 0.28 mm is located utilizing the protocol described in Table 6. The images were analyzed with a specialized software, AutoQALite (IrisQA, Frederick, MD), that calculates the modulation transfer function (MTF) from the images and returns the values of the spatial resolution in lp/cm at 50% and 10% of the MTF. The recommended acceptance level: the measured 50% and 10% of MTF is recommended to be within 0.5 lp/cm or ±10% of the value specified in the supplier's manual.^(10)^

**Table 6:** The acquisition parameters used for measurement of spatial resolution

*Technique*	*Axial*
kVp	120
mAs	250
Reconstruction kernel	Abdominal^a^
SFOV	Body/Large Body
DFOV	150 mm
Matrix size	512×512

^a^ Corresponding filters were Standard for GE VCT and GE Lightspeed 16, and FC13 for Toshiba Aquilon One.

#### C.4 Slice thickness

The slice thicknesses were measured in the CTP401 module of Catphan 500 and in the CTP404 of Catphan 504 or 600, where two pairs of 23° wire ramps, one parallel to the x‐axis and one parallel to the y‐axis, are located. The slice thickness of an image is defined as the full width half maximum (FWHM) of the slice sensitivity profile and was quantified as the FWHM length of any of the four ramp wires and multiplied by 0.42.^(28)^ The scan parameters used are shown in Table 7. These measurements were done automatically using specialized analysis software named AutoQALite. The recommended acceptance level for this test is: slice width >2 mm: maximum deviation ±1 mm, 1 mm<slice width<2 mm:deviation±50%, slice width<1 mm:maximum deviation±0.5 mm.^(10)^

**Table 7:** The acquisition parameters used for measurement of slice thickness

*Technique*	*Axial*
kVp	120
mAs	250
Reconstruction kernel	Standard
SFOV	Body/Large Body
DFOV	210 mm
Matrix size	512×512

### D. Data analysis

The images used for all image quality tests were burned on CDs as DICOM files, and were accessed and selected using an in‐house made MATLAB (MathWorks Inc., Natick, MA) program which allows users to determine whether the images were correctly positioned and of adequate quality. All the noise calculations were performed in this software. After the image selection, all the uniformity analysis, CT numbers and linearity analysis, and the MTF and slice thickness tests were performed using the software AutoQALite, which is specially developed for analysis of CT phantom images such as those used in this study. All images were assessed on stand‐alone computer. A 95% level of significance used in this study.

## III. RESULTS

### A. Uniformity

**Figure 1:**
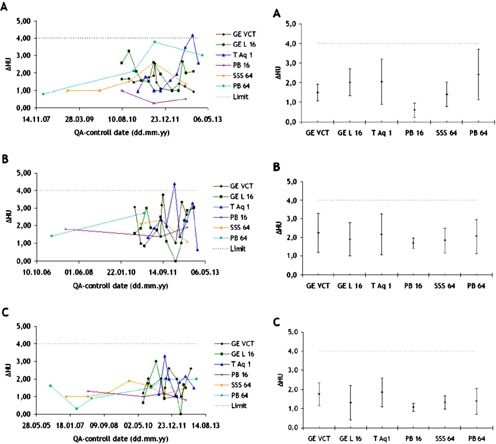
Uniformity measured in images acquired with (a) body, (b) head, and (c) helical protocols in the six scanners. The left panels correspond to the absolute uniformity values measured throughout time, whereas the right panels are time‐averaged values with their respective standard deviation. The dotted lines at 4 HU indicate the limit for an acceptable uniformity measurement.

The uniformity in CT images acquired in the homogeneous module of Catphan 500/504/600 was measured as defined above for each QA‐control. The results from these tests are shown in Fig. 1, where temporal changes in the uniformity are shown in the left panels and time‐averaged uniformity values with their respective standard deviations are presented in the right panels; in both cases, the A, B, and C indexes designate the results acquired with body, head, and helical protocols, respectively. It was observed that the uniformity values were within the advised limit (ΔHU≤4)^(10)^ for all measurements, with two exceptions in the Toshiba Aquilon One scanner. The first of these failures occurred due to unknown reasons in an image generated using a head protocol in February 2012 which yielded in a measurement of ΔHU=4.38, whereas the second failure, which happened in November 2012 using a body protocol that gave an output of ΔHU=4.19, was related to a ring artifact in the image. Afterwards, service engineers from Toshiba checked the CT scanner and did calibrations to improve the system. Overall, it was observed that, in spite of relatively large variations in uniformity values from one measurement to the next, these were shown to be acceptable. Moreover, the results show generally improved uniformity and smaller variations when the images were acquired by utilizing a helical protocol.

### B. Noise

**Figure 2:**
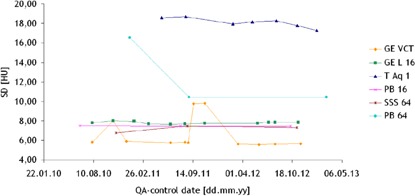
Noise measured in images acquired with a body protocol in the six scanners throughout time. The images were taken using Catphan phantoms in all the scanners with the exception of Toshiba Aquilon ONE, where the images were taken in a water phantom supplied by the manufacturer.

Noise, the average standard deviation values for all of the slices measured throughout time, using abdominal reconstruction kernels, is shown in Fig. 2. The main purpose of this figure is to present the temporal variations in this parameter for each scanner separately, independently from the others, since differences in acquisition protocols which will have a strong influence on noise. In the case of the GE VCT scanner, an abrupt variation in the SD measurements, ranging from 5.75 to 9.75 HU within a period of 20 days in the period August–September 2011 was observed. In the period from October 2012 to March 2013, the SD measurements were decreased from SD 9.81 to SD 5.61 HU. For the Philips CT scanners, the measurements were not performed as often, still a decrease in the measured SD values of Δ6.1 HU in the period of January–August 2011 was registered.

**Figure 3:**
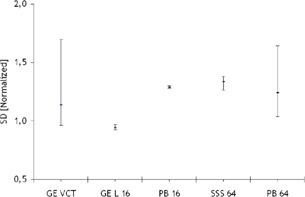
Normalized noise calculated from measurements in images acquired with a body protocol on the five scanners where the Catphan phantoms were utilized for this test. Error bars indicate the range in the measurements.

In order to make the results as comparable as possibly achievable, the noise values were normalized with respect to the slice thickness, and taking in consideration the CTDI for the normalized slice thickness, as expressed in Eq. (2). The averages of the normalized noise values for each of the CT scanners where noise was measured in a Catphan phantom are shown in Fig. 3, where the error bars depict the range in the calculations.

Normalization of noise resulted in lowest values of this parameter for GE Lighstpeed 16 and highest for Siemens Somatom Sensation 64. These also yielded in a broader distribution of data points for GE Lighstpeed VCT with respect to Philips Brilliance 64 due to wider slice reconstruction thickness. It is important to remark that this is purely a theoretical approach, where the effects of the differences in the reconstruction kernels implemented by the manufacturers have been neglected.

### C. Linearity and CT number

**Figure 4:**
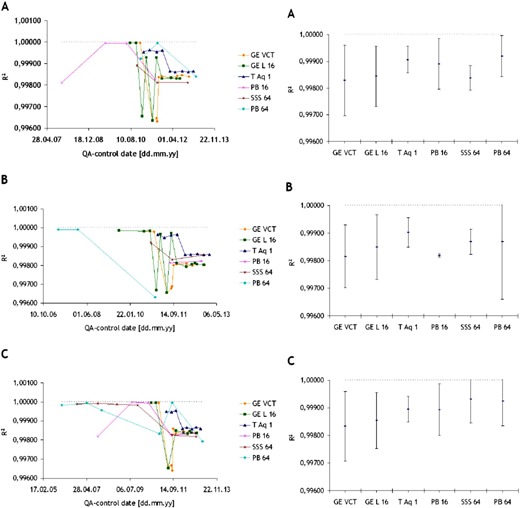
Linearity measured in images acquired with (a) body, (c) head, and (c) helical protocols. Left panels correspond to the linear coefficient values $R^{2}$ measured throughout time, and right panels to averaged values with their respective standard deviation. The dotted lines represent the highest achievable value for $R^{2}$ (1.0).

The output from the linearity tests is shown in Fig. 4, where the measured linearity over time are presented in the left panels, and the averaged values with corresponding standard deviation are shown in the right panels. In both cases, the letters A, B, and C indicate that the images for this test were acquired by using axial body, axial head, and helical protocols, respectively. Left panels correspond to the linear coefficient values $R^{2}$ measured in time, and right panels to averaged values with their respective standard deviation.

The results consistently satisfied the requirements for this test ($R^{2}\geq 0.98$) for all CT scanners. Nevertheless, it was possible to observe different amplitudes in the distribution of the data points, which appeared particularly higher for head protocols.

**Figure 5:**
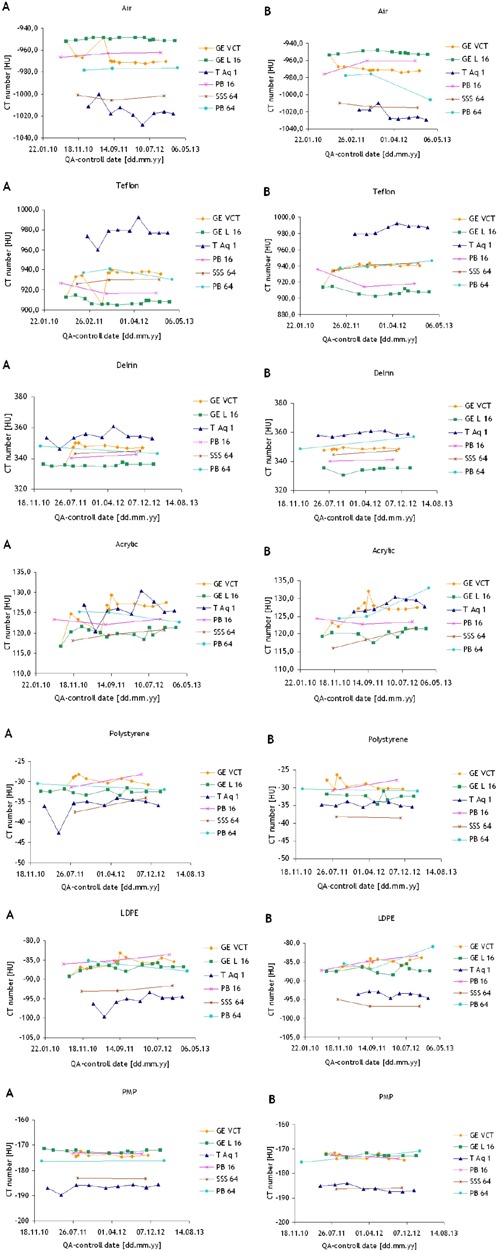
CT numbers measured for different sensitometric inserts acquired with (a) body and (b) helical protocols throughout time. The dotted line in each plot represents the expected HU value corresponding to the material of the sensitometric insert.

**Figure 6:**
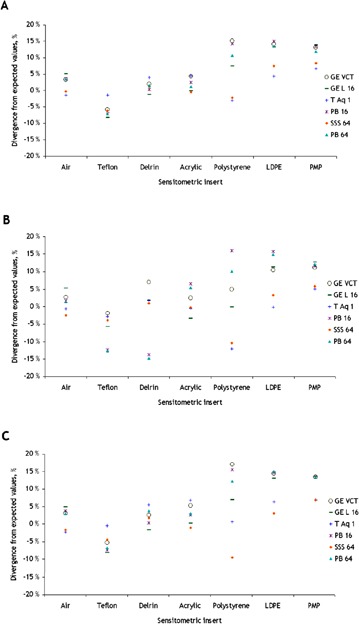
Time‐averaged percentage of divergence from nominal values of CT number in each sensitometric insert in images acquired with (a) body, (b) head, and (c) helical protocol for the different CT scanners.

The CT numbers in HU for each sensitometric insert obtained in our QA test are shown in Fig. 5, where the left panels indexed with the letter A, correspond to the images acquired with axial body protocols, and the right panels, marked with letter B, corresponds to helical protocols. Our data revealed that Toshiba Aquilon One registered higher values when measuring the HU of a positive sensitometric insert and lower values for negative CT number materials compared to the other scanners. The opposite tendency was observed for the corresponding results for GE Lightspeed 16. A significant spread in the CT number measurements between different CT scanners was observed, especially for the materials with the highest and lowest expected CT numbers for all the sensitometric inserts used in our tests. This phenomenon appeared independently of scan techniques used. For example, in the case of air (expected ‐1000 HU), average differences of ~65 and ~72 HU were found between the measurements for GE Lightspeed 16 and Toshiba Aquilon One, respectively, whereas for teflon (expected 990 HU) the average differences between these two scanners were ~69 and ~77 HU, respectively. Nevertheless, as it can be observed in Fig. 6, the average percentage of divergence from expected values was very low for these materials (≤±10%). This figure also shows that air, acrylic, and PMP yielded in more consistent divergence percentages from expected CT number values between the scanners, regardless of using abdominal, head or helical protocols. teflon and delrin resulted in consistent measurements as axial body and helical protocols were used, but returned larger variations as the head protocols were used. In the case of polystyrene and LDPE, in spite of registering absolute variations of less than ±10 HU for all the scanners, it was observed that the average percentage deviation from the expected values yielded in a very large range, for all protocols.

### D. Spatial resolution

The evaluation of the spatial resolution was performed based upon the results from three of the CT scanners (GE Lightspeed VCT, GE Lightspeed 16, and Toshiba Aquilon One) due to significant differences in the frequency of the tests and utilized methods in the other three scanners.

**Figure 7:**
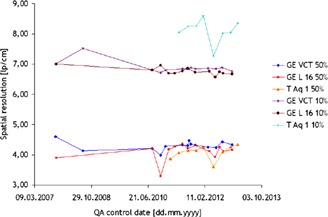
Spatial resolution at 50% and 10% of the modulation transfer function measured in images taken with three different CT scanners throughout time.

**Figure 8:**
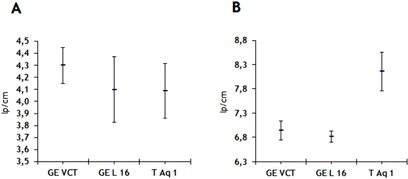
Spatial resolution at (a) 50% and (b) 10% of the modulation transfer function measured in images acquired with three different commercial CT scanners.

The measurements of this parameter performed by AutoQALite for each QA control over time are shown in Fig. 7. The curves on top of this figure represent the measured spatial resolution at 10% of the modulation transfer function (MTF), whereas the curves on the bottom render the spatial resolution at 50% of the MTF for the same CT scanners. It was observed that both the GE scanners performed in compliance to the recommendations according to international standards (IEC 2004, IEC 2006) (baseline value given by vendor ±10% lp/cm at acceptance test, and ±15% lp/cm for constancy tests) with one exception, GE Lightspeed 16, where the spatial resolution at 50% of the MTF was ~21% lower than the expected value when using the standard reconstruction algorithm (=4.20 lp/cm). In the case of Toshiba Aquilon ONE, there are no baseline values for the spatial resolution when using the FC13 reconstruction filter. However, it was observed that at 50% of the MTF the obtained results were just slightly lower than those for the two GE scanners, whereas at 10% of the MTF these appeared to be 1−1.5 lp/cm higher. The plots A and B of Fig. 8 show the average values of the spatial resolution at 50% and 10% of the MTF, respectively, with error bars symbolizing the standard deviation in the measurement.

### E. Slice thickness

**Figure 9:**
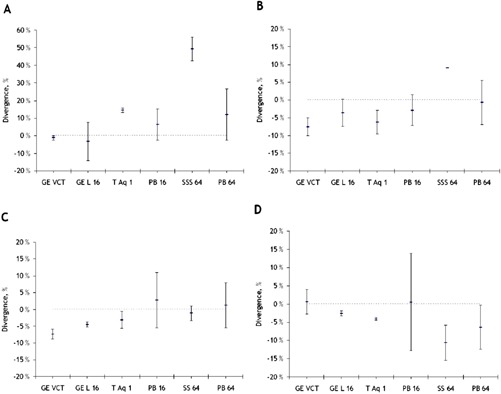
Interscanner divergence from nominal slice thickness values in slices in the intervals: (a) 0.5 to 0.625 mm, (b) 1.0 to 1.5 mm, (c) 2.4 to 3.25 mm, and (d) 4.0 to 5.0 mm. The dotted are placed as reference and correspond to a deviation of 0%.

Average measured divergence from nominal slice thickness for each scanner is shown in Fig. 9. Letters A, B, C, and D indicate nominal slice thicknesses of 0.5 mm−0.625 mm, 1.0 mm−1.5 mm, 2.4 mm−3.25 mm, and 4.0 mm−5.0 mm, respectively. For slices <1.0 mm, a large positive deviation from nominal values was measured for the Siemens scanner, and also for the Philips scanners the deviation was in the positive direction. For slices >1.0 mm, most scanners produce reconstructed slice thicknesses thinner than nominal values. Variability is large for the Philips scanners, and relatively small for the GE and Toshiba scanners.

## IV. DISCUSSION

The radiation protection authorities demand 1) periodical quality assurance tests with respect to image quality and radiation dose, and 2) optimization of all examination protocols with respect to image quality and radiation dose. The aim of this study was to evaluate different recommended image quality assurance tests over time.

Our results indicate that the uniformity is approximately the same over time, and that there is minor drifting for the results through time, so it might not be necessary to measure uniformity more often than once a year, if no artifacts or other problems with the scanner are reported.

Image noise affects low‐contrast detectability, especially in the case of detection of small low‐contrast lesions, such as liver metastasis. For the noise measurements, there were only minor deviations between the acceptance tests and annual test over time for the same scanners, except for the cases of GE Lightspeed VCT and Philips Brilliance 64. In general, the 16 slice CT scanners presented the smallest range in noise measurements in these tests, indicating that the measurements are more stable for the smaller detectors.

HUs are widely used in diagnostics.^(29)^ A number of papers describe possible methods for using HU measurements for diagnostic purposes, and describe recommendation for tissue characteristics, tissue differentiation and threshold values for malign and benign masses, for example in adrenal glands. Our results show a significant variation in the measurements of HU for different object densities between different CT scanners and also over time for the same scanner. The radiologists should be aware of this as long as HU are used for quantification and tissue characterizing for diagnostic purposes. Based on the results from our study, we recommend that HU should be used carefully for diagnostic purposes.

The spatial resolution is important for detection of small objects with high contrast to background, for example iodine‐enhanced vessels or small bone structures. Our results showed that the spatial resolution was not differing much over time. This result was expected, since the spatial resolution is most dependent on the detector element size, reconstruction matrix and DFOV, scanner geometry, focus size, and reconstruction algorithm used. There is no reason to suspect deviations in the spatial resolution over time for CT scanners, unless there is tube upgrade or detector upgrade or the reconstruction algorithms have been changed.

There are limitations in this study. First of all, the data presented are measured over time on different CT scanners, with different Catphan phantoms. The scan techniques used were as comparable as possible for all scanners, yet there are differences in collimations, reconstruction algorithms, and spectral energy that will influence the results. The data are not always useful for comparison between scanners, but are valuable for monitoring deterioration over time for each specific scanner and also to compare the deterioration between scanners.

As far as possible, the same Catphan phantom was used for the same CT scanners over time, but different phantoms were used for the measurements on different CT scanners. Interphantom differences may occur, and also intraphantom differences may occur over time. Still, the international recommendations and the CT vendors' technical specifications are based on the assumption that there are no interphantom or intraphantom differences. Evaluating possible interphantom and intraphantom variations was not the scope of this study, but should be tested carefully in the future.

Based on the results from this study, it is clear that some tests should be performed more often than others. Dose measurements have not been a topic in this study, but frequencies with respect to these measurements should also be considered as part of the total QA regime.

## V. CONCLUSIONS

The results from this study indicate that there is minor drifting in image noise, uniformity and in spatial resolution over time for CT scanners, independent of vendors. The HU for different object densities vary between different CT scanner vendors and models, and over time within the same CT scanner. Future tests of interphantom and intraphantom variations along with inclusion of more CT scanners are necessary to establish recommendations for robust methodology, baselines, and test frequencies for QA controls of CT systems independent of model and vendor.
